# Role of Platelet-Rich Plasma in the Management of Non-obstructive Azoospermia

**DOI:** 10.7759/cureus.69387

**Published:** 2024-09-14

**Authors:** Badr Alharbi

**Affiliations:** 1 Department of Surgery, College of Medicine, Qassim University, Buraydah, SAU

**Keywords:** assisted reproductive techniques, azoospermia, male infertility, platelet-rich plasma, spermatogenesis

## Abstract

Non-obstructive azoospermia (NOA) is a common cause of infertility in males, which is characterized by the absence of sperm in the ejaculate, resulting from impaired spermatogenesis. The primary therapeutic approaches for the management of NOA include testicular sperm extraction, varicocelectomy in case of clinical varicoceles, and hormonal manipulation. While traditional treatments are found to have a limited role in the management of NOA, recent studies have explored the potential of platelet-rich plasma (PRP) as a safer and promising therapeutic option. Autologous PRP preparations are derived from the patient's blood and comprise growth factors and cytokines, promoting tissue repair and regeneration. PRP has a wide range of applications in the medical field, including managing infertility in males and females. This literature review aimed to evaluate the existing evidence on the efficacy and safety of PRP in the management of NOA. After a thorough review of relevant data from observational and experimental studies, the findings of this study suggested that PRP may positively influence spermatogenesis, sperm quality, DNA integrity, and sperm retrieval during assisted reproductive procedures. Further research is needed to establish the optimal PRP preparation, administration method, and long-term benefits. The newer studies shall include a diverse patient population and employ long-term follow-up to assess the durability of any positive effects of PRP treatment. The growing body of evidence regarding the therapeutic potential for NOA in humans offers greater opportunities for men seeking fertility treatment, informing clinical practice, and optimizing the use of PRP.

## Introduction and background

Azoospermia is a disorder whereby the ejaculates of a man and his semen contain no sperm. This could be the result of the man not producing sperm or of the sperm being prevented from reaching the semen [1]. Azoospermia, regardless of its cause, inevitably leads to infertility. Global estimates indicate that azoospermia is diagnosed in approximately 1% of males of reproductive age and up to 10% of men presenting with infertility [2,3].

Azoospermia is categorized into obstructive and non-obstructive forms. This distinction has important clinical implications, as it significantly influences patient management strategies and ultimately affects treatment outcomes [3]. Obstructive azoospermia (OA) is a condition marked by the absence of spermatozoa in the ejaculate despite normal spermatogenesis. This disorder is a prevalent contributor to male infertility and can arise from a range of etiological factors, such as acquired pathological conditions, congenital anomalies, or iatrogenic injury [4]. Non-obstructive azoospermia (NOA) is a condition characterized by the absence of sperm in the ejaculate, resulting from the inability to produce sperm. It is considered the most severe type of male infertility [5]. The significant impairment in sperm production observed in patients with NOA is frequently caused by either primary testicular failure, primarily affecting spermatogenic cells (referred to as spermatogenic failure or STF), or dysfunction of the hypothalamus-pituitary-gonadal axis (known as hypogonadotropic hypogonadism or HH). Therefore, the abbreviations STF and HH are used to differentiate between these types of NOA, as appropriate [6]. It is crucial to differentiate between STF and HH because STF is associated with severe and incurable conditions, while HH can be successfully managed with gonadotropin therapy [7]. On the other hand, OA occurs due to a physical blockage in the male reproductive tract, such as the vas deferens, epididymis, or ejaculatory duct [8]. Unlike NOA, sperm production is maintained in OA, and both reconstructive surgeries as well as sperm recovery are generally extremely successful in OA patients [9,10].

The cause of NOA is the inability of the testes to produce sperm, either due to insufficient production of gonadotropins or inherent testicular dysfunction. The utilization of a comprehensive history, thorough physical examination, hormonal assessment, and genetic testing establish the diagnosis. The presence of factors such as previous anticancer chemotherapy or undescended testis raises concerns that the diagnosis may be related to spermatogenesis failure. Identifying the medications taken by the patient is crucial, as certain drugs, such as steroids and 5α‐reductase inhibitors, might negatively affect the production of sperm [11,12]. The assessment of the development of secondary sexual traits is based on the Tanner phases. Inadequate growth of the genitalia or pubic hair indicates the existence of hypogonadism. Accurate assessment of testicular size with an orchidometer or ultrasonography is crucial for diagnosing NOA. The size of the testes may serve as an indicator for the degree of spermatogenesis. Consequently, smaller testes imply a diminished likelihood of success in this process. Individuals diagnosed with non-obstructive azoospermia (NOA) typically exhibit testes with a volume below 15 cc, accompanied by a flattened epididymis [13].

Additional tests, including karyotyping and genetic analysis, are carried out after identifying NOA. According to reports, 13.7% of azoospermia patients have an aberrant karyotype [5]. Kallmann syndrome, characterized by hypogonadotropic hypogonadism with anosmia, is caused by several genetic abnormalities, including fibroblast growth factor receptor 1 (FGFR1) and KAL1 [14,15]. Mutations in the androgen receptor (AR) gene, located on the X chromosome, have been identified as a primary cause of moderate to severe androgen insensitivity syndrome (AIS), resulting in diminished androgen responsiveness and subsequent disruptions in male sexual differentiation [16]. Numerous genes located on the X chromosome are known to function exclusively on the testis and are crucial for meiosis [17]. Testing for azoospermia factor (AZF), which has three sub-regions (AZFa, AZFb, and AZFc) and is found on the long arm of the Y chromosome (Yq), is the most common and important genetic test for NOA [18]. 

## Review

Prevalence of azoospermia

One in six couples experience infertility, with male factor infertility being identified as a contributing factor in 50% of instances [19]. A diagnosis of azoospermia is found in 10 to 15% of infertile males and around 1% of all men [20]. According to [21], approximately 1% of males have infertility due to NOA, making up 10-15% of cases, and it is one of the main reasons for male infertility. Research in epidemiology found that oligozoospermia was present in 20% of 8,518 male partners of infertile couples. However, the study could only determine the cause of oligozoospermia in 25% of the instances [22]. Similarly, a separate study including 26,091 male partners of infertile couples found that 49% of them had oligozoospermia, whereas 11% had NOA. Only 28% of these couples achieved the diagnosis. Therefore, a significant proportion of these couples, up to 72%, had an unknown cause [23]. This discovery emphasizes the need for research that clarifies the cause of spermatogenic failure. With the incidence of spermatogenic failure expected to increase rapidly, the need to address this issue is becoming more urgent. Meta-regression findings further confirm a global trend of declining sperm concentration and count [24,25].

An examination of the TEX11, NR5A1, and DMRT1 genes in 80 individuals with NOA, normal chromosomal structure, and no Yq microdeletions revealed likely harmful mutations in four patients (5% of the whole group), resulting in a diagnostic rate increase of up to 25% [23]. In addition, examining a group of 15 genes in the blood samples of 25 patients with seemingly unexplained low sperm count or NOA resulted in the detection of harmful variations in the NR5A1 and TEX11 genes in three patients (12.0%) [25].

Pathophysiology of azoospermia

NOA encompasses three main categories: primary testicular failure, secondary testicular failure, and cases with an incomplete or ambiguous presentation of testicular failure. The first form is characterized by elevated levels of luteinizing hormone (LH) and follicle-stimulating hormone (FSH), small testes, and affects up to 10% of men with infertility. The second form refers to congenital hypogonadotropic hypogonadism, which is characterized by decreased levels of LH and FSH, as well as small testes. The third category encompasses cases with an ambiguous presentation of testicular dysfunction, characterized by disparate hormone and testicular volume profiles, including elevated FSH with normal testicular size, normal FSH with testicular atrophy, or normal FSH with preserved testicular volume. Notably, maturation arrest, a condition often associated with normal FSH levels and testicular size, can also be linked to underlying genetic anomalies, such as MLH1 mutations, marked by diminished recombination frequencies. Measurement of serum testosterone, LH, FSH, and prolactin levels may be used to differentiate between cases of NOA and OA. NOA may arise as a consequence of previous hazardous exposures, such as chemotherapy or radiation, or due to a history of aberrant growth, cryptorchidism, or big varicoceles [26,27]. 

Hypogonadotropic hypogonadism (HH) is a disorder characterized by low blood testosterone produced by a reduction in pituitary production of FSH and LH. HH might be inherited, acquired, or congenital. Congenital syndromes, including Prader-Willi, Laurence-Moon, and Kallmann syndromes, are traditionally syndromic. When a pituitary tumor, radiation treatment, or trauma destroys normal pituitary function, HH often occurs. Excess exogenous androgens or steroids may also cause acquired HH [28]. Infertility may also result from hyperprolactinemia because it prevents gonadotropin-releasing hormone (GnRH) from being secreted from the hypothalamus and because it prevents LH from attaching directly to the Leydig cells in the testis. Kallmann syndrome is the term for congenital HH, which usually coexists with anosmia. It may be passed down genetically via a variety of pathways, including autosomal defects and X-linked sex chromosomes [29]. Abnormal secondary sexual traits, such as micropenis and gynecomastia, are caused by hypogonadism. The principal anomaly is a reduction in hypothalamic GnRH release, which leads to a drop in pituitary gonadotropin levels (FSH and LH). HH is one rare cause of NOA that consistently responds to medicinal therapy [30].

Exogenous androgen excess or tumors of the pituitary, adrenal, or testicles can also result in the medically treated inhibition of spermatogenesis and NOA. This is a kind of HH in which spermatogenesis recovers when excess steroids are suppressed, and feedback inhibition on healthy pulsatile gonadotropin production is generated [31]. Congenital adrenal hyperplasia is a pediatric condition, and a significant proportion of these individuals will manifest infertility in adulthood. Infertility is prevalent when there are accompanying testicular adrenal rest tumors (TARTs) [32]. Increasing evidence suggests that TEX11 and TEX15 mutations are present in infertile males with NOA and meiotic arrest, similar to the mutant mice model [33,34]. Additionally, submicroscopic copy-number variants (CNVs) on the autosomes and X chromosome may have a role in NOA [35]. The presence of hypermethylation in the promoter region of the SOX30 gene leads to the suppression of its production in NOA. Additionally, the reduction in the level of SOX30 is correlated with the severity of NOA illness [36]. 

Management of non-obstructive azoospermia 

The primary treatment approaches for NOA include testicular sperm extraction (TESE), surgical ligation of clinical varicocele for patients with varicocele, gonadotropin replacement medication for those with HH, and other therapeutic methods such as fine needle aspiration (FNA) mapping before TESE [37]. Varicocelectomy is frequently performed in clinical varicoceles in order to enhance spermatogenesis in NOA patients. A comprehensive analysis of 16 observational studies revealed that the frequency of sperm presence in the ejaculate following varicocelectomy was 27.3%. Furthermore, the success rate of sperm retrieval by micro-TESE was greater in the varicocelectomy group compared to the untreated varicocele group (48.9% vs. 32.1%) [38]. A survey conducted across various medical institutes in Japan found that the administration of a combination of hCG and recombinant human FSH led to the presence of sperm in the ejaculate in 88.6% of the patients [39]. 

Platelet-rich plasma (PRP) is a concentrated form of platelets obtained by centrifuging a tiny amount of plasma. It is also referred to as autologous platelet gel, plasma rich in growth factors (PRGFs), and platelet concentrate (PC) [40]. The platelets transport around varieties of protein molecules, including cytokines, hormones, and chemo-attractants [41]. Upon activation, platelets produce a variety of physiologically active proteins that promote cell proliferation, growth, and differentiation [42]. Autologous platelet concentrates, which have a concentration three to seven times higher than the normal physiological level, are being utilized in several medical disciplines, such as orthopedics, dermatology, stomatology, sports medicine, and reproduction. These concentrates have shown significant positive results in these domains [43,44]. A multitude of components present in PRP have been demonstrated to exert a substantial beneficial impact on both the quality and functionality of sperm. For example, it has been discovered that transforming growth factor (TGF)-β and vascular endothelial growth factor (VEGF) can improve sperm motility [45,46], while zinc and calcium ions are intimately associated with sperm capacitation and acrosome response [47]. 

Therapeutic benefits of platelet-rich plasma 

PRP has been successfully utilized in reproductive biology to improve semen functionality and quality. It has been included in both fresh [48] and cryopreserved semen of various species, including bucks [49], rams [50], buffalo bulls [51], and humans [52]. Furthermore, PRP has been directly applied to the testes of bucks [53], resulting in improved sperm functionality and quality. In men, PRP has been utilized to address fertility issues caused by NOA [54]. Research has shown that PRP positively impacts reproductive health, as demonstrated by its ability to enhance characteristics of frozen-thawed sperm and testicular function in rabbits, as well as sperm morphology and quantity in rats. Notably, PRP also facilitates testicular tissue regeneration in rats and maintains the equilibrium of sex hormones. Moreover, its antioxidant properties help reduce oxidative stress and protect rat testicular tissues from injury, highlighting its potential as a therapeutic strategy for reproductive medicine [55].

An investigation on the impact of PRP on sperm physiology demonstrated that semen samples exposed to PRP for five minutes exhibited notably enhanced motility and morphometric characteristics. According to this study, the results were attained by the action of growth factors that were produced by secretory granules in the sample [48]. Another investigation demonstrated that the addition of 10% PRP to semen samples resulted in enhanced sperm quality and function, both in equilibrated and frozen-thawed specimens. Moreover, the PRP-supplemented groups exhibited increased antioxidant enzyme activities compared to the control group, particularly in the thawed semen samples, indicating a beneficial effect of PRP in mitigating oxidative stress and promoting sperm viability [56]. Multiple studies have shown a significant association between sperm DNA damage and male infertility [57]. In separate research, a total of 100 semen samples were used. After semen analysis, the samples were split into two equal portions: one served as a control without autologous PRP supplementation, while the other received a 2% concentration of autologous PRP. The results showed a marked decrease in the DNA fragmentation index in the PRP-treated group, achieving statistical significance (p < 0.001). The average integrity of sperm DNA was decreased with the addition of PRP, with a mean value of 33.85±16.73 compared to 38.55±16.64 (mean±standard error) [58]. Another study found that adding 5% PRP increased the progressive motility of sperm, as well as their vitality and membrane integrity following cryopreservation. This improvement was statistically significant (P<0.05). PRP supplementation of post-thawed spermatozoa resulted in a significant attenuation of ROS production, accompanied by a recovery of mitochondrial membrane potential and a reduction in DNA fragmentation [52].

After administering autologous PRP, 15 out of the 50 patients showed therapeutic results within a period of three to four months. The findings demonstrated a substantial disparity in the levels of FSH and LH hormones after the administration of PRP injection, with a p-value of 0.001 indicating strong statistical significance. PRP might assist infertile guys with the structural and functional abnormalities of their testes [59]. After treating the samples with PRP, a significant enhancement with a concentration of 2% PRP was observed. Therefore, this concentration is considered the most effective for positively impacting sperm parameters. Non-stressed and stressed (with H_2_O_2_) spermatozoa that were treated with 2% PRP exhibited a noteworthy enhancement in both progressive and total motility. Additionally, there was a reduction in the number of cells that tested positive for ROS, DNA fragmentation, vacuolization, and cell death compared to the untreated group. Furthermore, the application of 2% PRP therapy improved sperm parameters and inhibited cell death in spermatozoa exposed to H_2_O_2_, as compared to semen that was recently collected [60]. In a separate trial, a total of seventy-one individuals were administered half ml of PRP in each testicle. The PRP was obtained by subjecting the patient's own blood to centrifugation. The patients' FNA parameters and FSH levels were assessed both before and during the surgery. Several NOA instances had regular spermatogenesis. The post-procedure FSH level was greater than the pre-procedural FSH level. Patients with spermatocytes mentioned in the first FNA report had a decreased proportion of azoospermia compared to their counterparts [54]. A comparative analysis was conducted to examine sperm concentration, motility, and morphology in the ejaculation of 33 individuals who received PRP therapy. This study compared the PRP group to a control group of 35 men who did not undergo PRP therapy within a six-month timeframe after starting treatment. After four months of treatment with PRP, sperm concentration and motility showed a rise in 18 out of 33 males, as compared to their initial baseline measurements. In contrast to the PRP group, the group of men who did not get PRP therapy did not see any changes in sperm parameters compared to the initial measurements over four months [61]. Previous research has shown that PRP has an antioxidant impact, which inhibits oxidative stress by reducing the generation of pro-inflammatory cytokines and boosting antioxidant defenses that provide a protective impact on the male reproductive system. According to these findings, men who have aberrant semen parameters may benefit from treating their semen samples with PRP [62].

PRP is an efficient and minimally invasive approach to achieve natural levels of autologous growth factors. PRP is produced by centrifuging autologous blood and separating plasma. The remaining buffy coat protein is composed of high levels of platelets. Platelets mediate tissue repair and angiogenesis while promoting wound healing as PRP suppresses excessive inflammation [63]. 

The regenerative capacity of biologically active constituents in PRP and the safety of PRP preparations confer its use in a wide range of clinical applications. PRP-based growth factors include platelet-derived growth factor (PDGF), VEGF, fibroblast growth factor (FGF), transforming growth factor (TGF), and epidermal growth factor (EGF) [64]. Other cytokines and growth factors include hepatocyte growth factor, insulin-like growth factor (IGF)-1, IGF-2, interleukin-8 (IL-8), matrix metalloproteinase (MMP)-2, and MMP-9 [65]. 

PRP injection therapy is associated with high safety levels across various clinical applications, including the treatment of osteoarthritis and aging skin; however, PRP preparations may have decreased safety compared to pure autologous PRP [66-68]. Moreover, the application of PRP preparations in spinal diseases raises disputes, prompting the production of safer PRP preparations [69]. As demonstrated in animal models and human studies, PRP is a safe and effective method for improving male infertility [61,70]. 

*Platelet-Rich Plasma and Spermatogenesis* 

Besides PRP treatment-mediated increases in the proliferation and differentiation of progenitor cells, PRP is also implicated in improving structural and functional testicular impairment [71]. PRP reduces ischemic reperfusion injury in the testing by decreasing the concentration of reactive oxygen species. PRP is also known to have a protective effect on the function of Leydig cells and Sertoli cells in the testis [55]. Saba et al. demonstrated that PRP treatment results in the renewal of testis microstructure and is also responsible for the regeneration of tubules, mediated by cytokines and growth factors in PRP [72]. Moreover, in both human and animal models, PRP mediates morpho-functional restoration of testes [54]. 

Various studies in the medical literature have demonstrated the role of PRP in improving the self-renewal of spermatogonial stem cells in humans. Khadivi et al. investigated the effect of PRP on the proliferation and preservation of spermatogonial stem cells in two-dimensional and three-dimensional cultures. The study findings indicated that the PRP scaffold provides a structure suitable for the proliferation of spermatogonial stem cells [73]. Likewise, the study conducted by Lestari et al. revealed that PRP could be used as an alternative to fetal bovine serum in maintaining the proliferation and differentiation of spermatogonial stem cells, which was positively correlated with the magnitude of spermatogenesis [74]. Al-Nasser et al. demonstrated that male patients with prior primary and secondary spermatogenesis had a lower degree of azoospermia after PRP treatment than males without pre-treatment spermatogenesis [54]. 

In addition to cytokines and growth factors in PRP with regenerative potential, PRP administration decreases inflammatory mediates and increases the concentration of anti-inflammatory mediators. Intratesticular administration of PRP is significantly associated with an increase in testosterone levels and improvement in Leydig cell dysfunction and testicular steroidogenesis [61,75,76]. Moreover, growth factors in PRP reduce the duration of different meiotic stages, shortening the time for spermatozoa formation [75]. Studies also demonstrated a statistically significant increase in the levels of FSH and LH following PRP treatment [54,59,76]. The increase in the levels of FSH is attributed to the effect of PRP on Sertoli cells in the testis [59]. Another mechanism by which PRP mediates spermatogenesis is by increasing the height and thickness of the testicular germinal layer, which comprises male gametes in different stages of development [77]. 

*Platelet-Rich Plasma and Sperm Quality* 

The male fertility indicators, also described as sperm quality parameters, are divided based on the structural and functional evaluation of sperm. The structural parameters include appearance, morphology, chromatin and plasma membrane integrity, and concentration. The functional parameters include acrosomal reaction, motility, and capacitation [78]. Decreased sperm count is the prevailing characteristic of male infertility [79].

The positive effects of PRP on sperm quality are linked to its rich content of growth factors, which stimulates cell growth and repair. At an optimized concentration, PRP has a protective effect on sperm quality. The study conducted by Nabavinia et al. demonstrated that PRP was associated with significantly high sperm viability and progressive motility. Additionally, PRP treatment reduces the concentration of abnormal sperm to a significant extent [80]. Autologous PRP treatment also improves membrane integrity, decreases DNA fragments, and restores mitochondrial membrane potential [52]. Men diagnosed with severe oligoasthenoteratozoospermia also experience positive outcomes with PRP treatment, as evidenced by improvements in sperm motility and the percentage of morphologically normal spermatozoa [61].

There are several mechanisms by which PRP improves sperm quality parameters. The biologically active constituents in PRP exert a positive cryoprotective effect on the motility and viability of the sperm. FGF in PRP escalates the phosphorylation of flagella and triggers the protein kinase B signaling pathways, increasing the progressive motility. Serotonin in PRP increases the curvilinear velocity of sperm. IGF-1 and nerve growth factor (NGF) in PRP improve sperm membrane integrity and the stability of DNA [52]. Autologous PRP has also been demonstrated to improve the functional sperm parameters in cases of hydrogen peroxide (H_2_O_2_)-induced oxidative stress [60]. 

*Platelet-Rich Plasma and DNA Integrity* 

Autologous PRP has been revealed to enhance the DNA integrity of human sperm and decrease the DNA fragmentation index. PRP has outperformed other treatment options regarding reduction in the fraction of sperm DNA fragmentation. This is mediated by autologous PRP's antioxidant and antiapoptotic properties, which inhibit reactive oxygen species and reduce the DNA fragmentation index. The Zn/Cu/superoxide dismutase enzyme in PRP, an important constituent of the reactive oxygen species scavenger system, decreases H_2_O_2_-mediated DNA fragmentation by minimizing lipid peroxidation. IGF-1 and VEGF in PRP are also critical to improvement in DNA integrity [58]. Animal-based research has also shown the role of PRP in improving gene expression and reducing the levels of DNA fragmentation in cryopreserved sperms, comparable to the levels of DNA fragmentation and gene expression before freezing. These studies have also indicated the effectiveness of PRP treatment in countering oxidative stress and enhancing sperm quality [81]. 

Platelet-Rich Plasma and Sperm Retrieval

The retrieval of sperms in NOA involves various surgical techniques, including testicular sperm aspiration and testicular sperm extraction. Unsuccessful microdissection testicular sperm extraction in NOA patients may prompt the administration of intratesticular injection of autologous PRP. Gudelci et al. demonstrated that intratesticular PRP injection improved sperm identification and sustained implantation in NOA patients with one failed microdissection testicular sperm extraction, whereas PRP had comparatively lesser effectiveness in NOA patients with two or greater failed microdissection testicular sperm extraction attempts [82]. The same study demonstrated that fertilization was successful with intracytoplasmic sperm injection (ICSI) in patients administered intratesticular PRP [82].

Supplementation of frozen-thawed human sperm with autologous PRP, as part of the cryopreservation process, is associated with a protective effect on the human sperm and concomitant improvement in sperm parameters [52]. A study investigating the relationship between cryopreserved sperm from infertile males and pregnancy outcomes with ICSI demonstrated that pregnancy outcomes were significantly reduced when cryopreserved sperms were used for ICSI in males with varicocele-related NOA [83]. PRP is also associated with positive pregnancy outcomes in the frozen embryo transfer approach by improving sperm quality and reducing DNA fragmentation [84]. Similar results are observed in using PRP for in vitro fertilization and intrauterine insemination in the case of abnormal sperm [62].

The underlying processes responsible for improved sperm cryopreservation include improvement in sperm function, sperm survival, and protection against oxidative stress [81]. Cryopreservation has several negative effects on sperm survival and motility, harming the initial sperm quality and the proportion of motile spermatozoa. Cryodamages also include alterations in the structure of acrosome, mitochondria, genetic composition, and protein profile [85]. Thawed semen samples treated with 5% PRP exhibit enhanced membrane integrity, decreased acrosomal alterations, increased superoxide dismutase activity, reduced lipid peroxidation, enhanced antioxidant potential, and improved progressive motility. Additionally, using 5% PRP is associated with higher fertilization potential and fertilization rates when employed during semen cryopreservation [81]. 

*Treatment Protocol of Platelet-Rich Plasma for Non-obstructive Azoospermia* 

Based on the treatment protocol employed in a study, 2-3 ml of autologous PRP is administered into each testis using a 21 gauge needle and a 5 ml syringe [86]. Dosimetry can be utilized to administer optimized PRP doses [73]. Intratesticular PRP administration takes place under local or sedation anesthesia in an operating room, whereby autologous PRP is given percutaneously with a butterfly needle. Autologous PRP is injected into the seminiferous tubules or interstitial space without causing tension in the tunica albuginea [82]. PRP can be combined with cryopreservation to improve spermatogenesis [87]. 

## Conclusions

In summary, autologous PRP or PRP preparation holds significant potential for treating NOA. According to current evidence, PRP enhances spermatogenesis and improves sperm quality, mediated by the higher concentration of growth factors and cytokines in PRP. PRP also increases the possibilities of sperm recovery in males diagnosed with NOA, exhibiting cryoprotective properties. Despite the existing studies about the safety, effectiveness, and outcomes of PRP in NOA, extensive and well-designed investigations are required to validate the clinical utility and identify the maximum therapeutic potential of the treatment modality. Notably, the ongoing Türkiye-based clinical trial, "The Effects of Intratesticular PRP Injection in Men with Azoospermia or Crytozoospermia" (NCT04237779), may potentially contribute to the topic significantly and provide additional insights.
